# Sick days glycaemic outcomes in a cohort of children and adolescents with type 1 diabetes using an AID system—A preliminary report

**DOI:** 10.1111/dom.16377

**Published:** 2025-04-02

**Authors:** Davide Tinti, Cecilia Nobili, Gioia Bettin, Enrica Roggero, Alessandra Bondanese, Michela Trada, Alessia Gerace, Luisa de Sanctis

**Affiliations:** ^1^ Center for Pediatric Diabetology A.O.U. Città della Salute e della Scienza di Torino Torino Italy; ^2^ Postgraduate School of Pediatrics University of Torino Torino Italy; ^3^ Department of Public Health and Pediatric Sciences University of Torino Torino Italy

**Keywords:** glycaemic control, insulin pump therapy, real‐world evidence, type 1 diabetes

## 1 BACKGROUND

Sick days management still represents a significant challenge in type 1 diabetes (T1D), often leading to glycaemic instability and an increased risk of complications such as diabetic ketoacidosis (DKA).^1^, ^2^, ^3^ Automated insulin delivery (AID) systems, such as the SmartGuard™ algorithm embedded in the MiniMed™ 780G (Northridge, CA, USA), provide dynamic, real‐time insulin adjustments that optimize glycaemic control in routine care for adults and children^4^, ^5^; for this reason, these systems have been increasingly used during the past years.^6^ They have also demonstrated effectiveness in special clinical scenarios, including pregnancy, delivery and menstrual phases.^7^, ^8^, ^9^ Regarding paediatric care, optimal outcomes were also shown when introduced at diabetes' onset.^10^ However, their performance during intercurrent illnesses remains unexplored. To the best of our knowledge, this is the first study to evaluate the performance of an AID system during intercurrent illnesses in a cohort of children and adolescents with T1D, all using the same technology, under real‐world conditions.

## 2 METHODS

This observational, prospective cohort study investigated glycaemic outcomes in a cohort of children with T1D using Medtronic Minimed™ 780G in a real‐world setting.

Eligible participants included children and adolescents aged 1 to 18 with a diagnosis of type 1 diabetes mellitus using Minimed™ 780G with activated SmartGuard™ algorithm. The only exclusion criterion was using the pump in manual mode. Families were recruited during routine visits at the Diabetology clinic of the S.S.D. Paediatric Endocrinology at the Regina Margherita Children's Hospital in Turin, Italy.

The Research Ethics Committee (protocol number 888.296) reviewed and approved the study, which was conducted in accordance with the Declaration of Helsinki, Good Clinical Practice and applicable local regulatory requirements. Before study initiation, all caregivers provided written informed consent.

Children with T1D were enrolled between November 2023 and April 2024. Families were provided with a questionnaire to be completed following episodes of intercurrent illness, available in both paper and electronic formats (via Google Forms). Families were instructed to complete the form over a 12‐month period whenever one or more symptoms persisted for at least 24 h, including rhinorrhea, fever, sore throat, ear pain, nausea, vomiting, diarrhoea, or other relevant conditions. Additionally, intercurrent illness was defined by the need for antibiotic and/or steroid treatment, hospitalization, or an emergency room stay of at least 8 h. The questionnaire collected detailed information on the onset, duration, symptoms, treatment and glycaemic management during illness; however, this preliminary report did not include treatment details. Insulin and glycaemic data were extracted from CareLink™ downloads for analysis.

Glycaemic and insulin data were analysed across three periods: 14 days before symptom onset (baseline), during the illness and 14 days after symptom resolution (recovery). For this preliminary report, only glycaemic and insulin data during the symptomatic period were analysed. Serious adverse events were defined as DKA and severe hypoglycaemia.

## 3 STATISTICAL ANALYSIS

All analyses were performed using The Jamovi Project software (Version 2.6.2.0, 2024). The glycaemic outcomes and insulin therapy data were compared using a paired *t*‐test when the normality assumption was met; if the normality assumption was not met, a Mann– Whitney *U* test was used instead. A chi‐squared test was performed for categorical variables. All data are presented as mean ± SD, and *p*‐values <0.05 were taken to indicate statistical significance.

## 4 RESULTS

Of the 113 participants, 35 experienced at least one illness episode, with 9 individuals reporting multiple events, resulting in 51 recorded cases. Among these, 22 events involved fever, 19 involved respiratory tract infections (including rhinitis, cough, sore throat, tracheitis and bronchitis), and 10 involved gastrointestinal symptoms such as nausea, vomiting and diarrhoea. No serious adverse events were observed. The mean duration of symptoms across all episodes was 3.24 days.

Baseline demographics are shown in Table 1.

**TABLE 1:** Baseline demographics and clinical characteristics.

	Participants (*n* = 113)
Age (years), mean (range)	9.8 (2–18)
Sex, *n* (%)	
Female	46 (40.7)
Male	67 (59.3)
Duration of diabetes (years), mean ± SD	3.7 ± 2.7
Duration of AID use (months), mean ± SD	31.1 ± 11.1
Baseline HbA_1c_ (mmol/mol, %), mean ± SD	52.3 ± 8.5, 6.9 ± 0.8
AID start at onset, *n* (%)	
Yes	80 (70.8)
No	33 (29.2)

Abbreviations: AID, Automatic Insulin Delivery.

The mean variations in Time in Range (TIR) between the baseline and illness periods were analysed: no statistically significant differences were found between the mean TIR during the symptomatic period (69.7 ± 12.9%) and baseline TIR (71 ± 9.8%, *p* = 0.157). A Chi‐squared test was conducted to investigate further the relationship between TIR variation (divided into four percentiles) and symptom type. Symptoms were categorized into two groups: those likely to increase glucose levels (e.g., fever, respiratory infections) and those likely to decrease glucose levels (e.g., gastrointestinal symptoms), showing a statistically significant association (*χ* ^2^ = 13.0, *p* = 0.005). These findings highlight the notable influence of symptom type on TIR variation during illness, enabling a sub‐analysis by symptom type. Baseline glucose metrics showed no statistically significant differences.

During sick days, the symptoms likely to lead to hyperglycaemia resulted in higher mean glucose levels (Δ +8.4 mg/dL, *p* = 0.003), reduced TIR (Δ −4.1%, *p* = 0.007), and increased TAR‐1 (Δ +2.6%, *p* = 0.030). Conversely, symptoms likely to lead to hypoglycaemia led to an improved TIR (Δ +8.5%, *p* = 0.047) and a decrease in TAR‐1 (Δ −7.8%, *p* = 0.014), despite a slight, non‐significant rise in hypoglycaemia metrics. Detailed information is shown in Table 2.

**TABLE 2:** Glucose metrics (values are expressed as mean ± standard deviation) during baseline, hypoglycemic illness and hyperglycaemic illness across three periods: baseline (2 weeks prior to symptoms), during symptoms leading to hyperglycaemia and hypoglycaemia and recovery (2 weeks after symptoms).

	Symptoms leading to hyperglycaemia					Symptoms leading to hypoglycaemia				
	Baseline	Illness	*p*‐value	Recovery	*p*‐value	Baseline	Illness	*p*‐value	Recovery	*p*‐value
Glucose metrics										
TAR‐2, %	6.0 ± 4.6	8.0 ± 6.2	**0.045** *	7.1 ± 5.2	0.178	4.5 ± 3.6	2.4 ± 3.3	0.161	5.7 ± 6.0	0.720
TAR‐1, %	20.2 ± 7.6	22.8 ± 7.4	**0.030** *	20.2 ± 6.4	0.756	19.7 ± 7.7	11.9 ± 7.7	**0.014** *	18.3 ± 6.6	0.678
TIR, %	71.1 ± 10.1	67.0 ± 11.8	**0.007** **	69.7 ± 10.0	0.241	72.1 ± 8.4	80.6 ± 11.5	**0.047** *	73.9 ± 11.5	0.554
TBR‐1, %	2.2 ± 2.1	1.7 ± 2.0	**0.031** *	2.5 ± 2.0	0.417	3.3 ± 1.8	4.0 ± 3.2	0.309	2.0 ± 1.4	0.056
TBR‐2%	0.4 ± 0.6	0.4 ± 0.9	0.782	0.5 ± 0.7	0.704	0.4 ± 0.5	1.1 ± 1.4	0.168	0.1 ± 0.3	0.149
Mean glucose, mg/dL	150.5 ± 17.6	158.9 ± 18.3	**0.003** **	152.5 ± 16.1	0.267	145.3 ± 15.9	133.8 ± 16.0	0.059	148.3 ± 18.5	0.953
CV, %	35.6 ± 5.8	34.8 ± 7.1	0.480	36.9 ± 6.5	**0.028** *	36.2 ± 5.0	31.5 ± 6.4	**0.049** *	36.2 ± 5.1	0.570
Insulin data										
Total daily dose, UI	21.1 ± 13.4	21.0 ± 13.5	0.946	22.3 ± 13.1	0.249	31.6 ± 16.4	24.0 ± 14.2	**0.027** *	28.8 ± 15.3	**0.012** *
Basal, %	39.4 ± 5.2	40.6 ± 7.3	0.181	39.2 ± 6.6	0.850	38.5 ± 4.1	46.2 ± 14.3	0.080	39.2 ± 5.4	0.905
Boluses, %	60.6 ± 5.2	59.4 ± 7.3	0.181	60.8 ± 6.6	0.850	61.4 ± 4.1	53.8 ± 14.3	0.080	60.8 ± 5.4	0.905
Auto‐correction, %	36.1 ± 12.4	41.6 ± 10.4	**<0.001** ***	36.4 ± 29.0	0.290	30.1 ± 6.3	33.6 ± 9.4	0.164	29.0 ± 7.1	0.086
Time in SmartGuard, %	99.5 ± 1.2	98.3 ± 5.1	0.207	98.5 ± 2.9	0.079	98.9 ± 1.6	98.7 ± 4.1	0.396	98.6 ± 2.2	0.710
Carbohydrates, g/day	159.6 ± 51.5	141.1 ± 58.6	**0.011** *	164.0 ± 52.6	0.602	205.0 ± 45.3	141.5 ± 59.4	**<0.001** ***	181.2 ± 32.2	0.086

*Note*: Glycaemic ranges include Time in Range (TIR, 70–180 mg/dL), Time Above Range‐1 (TAR‐1, 180–250 mg/dL), Time Above Range‐2 (TAR‐2, >250 mg/dL), Time Below Range‐1 (TBR‐1, 54–70 mg/dL) and Time Below Range‐2 (TBR‐2, <54 mg/dL). Statistical significance was evaluated using the Mann–Whitney *U* test. All values are compared to baseline values.

* *p* < 0.05;

** *p* < 0.01;

*** *p* < 0.001.

Carbohydrate (CHO) intake decreased significantly in both scenarios (*p* < 0.001 for symptoms leading to hyperglycaemia and *p* = 0.011 for symptoms leading to hypoglycaemia), and auto‐corrections increased during episodes involving symptoms leading to hyperglycaemia (*p* < 0.001). While insulin delivery metrics showed no significant changes in the former category (Δ −0.1 UI, *p* = 0.946), symptoms likely to lead to hypoglycaemia required a lower total daily dose (TDD) (Δ −7.6 UI, *p* = 0.027).

The SmartGuard™ algorithm remained highly active across all episodes, with no significant differences in the percentage of algorithm activation between baseline and symptomatic periods.

While evaluating glucose metrics and insulin delivery information in the recovery period, it is interesting to note that it can be possible to observe a complete return to baseline conditions.

## 5 CONCLUSIONS

This preliminary report evaluated the impact of intercurrent illnesses on glycaemic control in children and adolescents with type 1 diabetes using the SmartGuard™ algorithm. The findings showed global stability in TIR across weeks despite the age group and the impact of the intercurrent disease, suggesting that AID can effectively mitigate severe glycaemic excursions, even in illness. Notably, the time spent in SmartGuard™ was consistent across all conditions, demonstrating the system's reliability and the users' trust.

On another note, speculating on total insulin, in the case of illness reducing insulin requirements (e.g., gastroenteritis), the algorithm was effective in maintaining good values (TIR even improved without increasing the TBR), but this cannot be said for illness increasing insulin requirements (e.g., fever), where TIR decreased significantly. While the reduced CHO intake likely contributes to the observed reduction in TDD, it is plausible that the algorithm requires further optimization to address intercurrent illnesses better. Interestingly, these results partially contrast with current ISPAD guidelines, which suggest the eventual switching of AID systems to manual mode during intercurrent illnesses to allow direct control over insulin delivery.^1^ The algorithm's stable performance, especially during gastroenteritis, challenges this recommendation, suggesting it can provide great glycaemic control without requiring manual adjustments.

This study is limited by the focus on glycaemic outcomes only during the symptomatic period, which may introduce bias and limit generalizability. Additionally, the small sample size and imbalance between glucose‐increasing and glucose‐decreasing symptom groups may affect the robustness of our conclusions. Future studies should assess glycaemic trends over longer periods and in larger, more balanced cohorts to better understand the impact of intercurrent illnesses in children using AID systems.

In conclusion, these findings emphasize the potential of AID systems to support glycaemic control in challenging scenarios and highlight the need for continued research to optimise their use in real‐world clinical practice.

## AUTHOR CONTRIBUTIONS

D.T., C.N. and L.d.S. were involved in the conception, design and conduct of the study. G.B., C.N., E.R., A.B. and A.G. were involved in data collection. D.T., C.N. and L.d.S. analysed and interpreted the results. C.N. wrote the first draft of the manuscript, and all authors edited, reviewed and approved the final version of the manuscript. D.T. is the guarantor of this work and, as such, has full access to all the data in the study and takes responsibility for the integrity of the data and the accuracy of the data analysis.

## FUNDING INFORMATION

No external funding was provided concerning this study.

## CONFLICT OF INTEREST STATEMENT

All authors have no relevant conflict of interest to disclose.

## PEER REVIEW

The peer review history for this article is available at https://www.webofscience.com/api/gateway/wos/peer-review/10.1111/dom.16377.

## Data Availability

The data that support the findings of this study are available on request from the corresponding author.
